# Multimodal Preventive Trial for Alzheimer’s Disease: MIND-ADmini Pilot Trial Study Design and Progress

**DOI:** 10.14283/jpad.2022.4

**Published:** 2022-01-20

**Authors:** S. Sindi, C. Thunborg, A. Rosenberg, P. Andersen, S. Andrieu, L. M. Broersen, N. Coley, C. Couderc, C. Z. Duval, G. Faxen-Irving, G. Hagman, M. Hallikainen, K. Håkansson, J. Lehtisalo, N. Levak, F. Mangialasche, J. Pantel, E. Kekkonen, A. Rydström, A. Stigsdotter-Neely, A. Wimo, T. Ngandu, H. Soininen, T. Hartmann, A. Solomon, Miia Kivipelto

**Affiliations:** 1 Division of Clinical Geriatrics, Department of Neurobiology, Care Sciences and Society, Karolinska Institute, Soina, Sweden; 2 The Ageing Epidemiology (AGE) Research Unit, School of Public Health, Imperial College London, London, UK; 3 Theme Inflammation and Aging, Karolinska University Hospital, Stockholm, Sweden; 4 Department of Neurology, Institute of Clinical Medicine, University of Eastern Finland, Kuopio, Finland; 5 Department of Clinical Epidemiology and Public health, CHU de Toulouse, and INSERM, University of Toulouse, UMR1027 Toulouse, France; 6 Danone Nutricia Research, Advanced Medical Nutrition, Utrecht, The Netherlands; 7 German Institute for Dementia Prevention (DIDP), Saarland University, Homburg, Germany; 8 Institute of General Practice, Goethe University, Frankfurt a.M., Germany; 9 Population Health Unit, Department of Public Health and Welfare, Finnish Institute for Health and Welfare, Helsinki, Finland; 10 Department of Social and Psychological Studies, Karlstad University, Karlstad, Sweden; 11 Engineering Psychology, Luleå University of Technology, Luleå, Sweden; 12 Centre for Research & Development, Uppsala University/County Council of Gävleborg, Gävle, Sweden; 13 Department of Experimental Neurology, Medical Faculty, Saarland University, Homburg, Germany; 14 Stockholms Sjukhem, Research & Development Unit, Stockholm, Sweden; 15 Karolinska Institutet, Dept NVS, Division of Clinical Geriatrics, Center for Alzheimer Research QA32, Karolinska vägen 37 A, SE-171 64 Solna, Sweden

**Keywords:** Dementia, Alzheimer’s disease, prevention, lifestyle, intervention, multimodal, randomized controlled trial

## Abstract

**Background:**

Interventions simultaneously targeting multiple risk factors and mechanisms are most likely to be effective in preventing cognitive impairment. This was indicated in the Finnish Geriatric Intervention Study to Prevent Cognitive Impairment and Disability (FINGER) testing a multidomain lifestyle intervention among at-risk individuals. The importance of medical food at the early symptomatic disease stage, prodromal Alzheimer’s disease (AD), was emphasized in the LipiDiDiet trial. The feasibility and effects of multimodal interventions in prodromal AD are unclear.

**Objectives:**

To evaluate the feasibility of an adapted FINGER-based multimodal lifestyle intervention, with or without medical food, among individuals with prodromal AD.

**Methods:**

MIND-ADmini is a multinational proof-of-concept 6-month randomized controlled trial (RCT), with four trial sites (Sweden, Finland, Germany, France). The trial targeted individuals with prodromal AD defined using the International Working Group-1 criteria, and with vascular or lifestyle-related risk factors. The parallel-group RCT includes three arms: 1) multimodal lifestyle intervention (nutritional guidance, exercise, cognitive training, vascular/metabolic risk management and social stimulation); 2) multimodal lifestyle intervention+medical food (Fortasyn Connect); and 3) regular health advice/ care (control group). Primary outcomes are feasibility and adherence. Secondary outcomes are adherence to the individual intervention domains and healthy lifestyle changes.

**Results:**

Screening began on 28 September 2017 and was completed on 21 May 2019. Altogether 93 participants were randomized and enrolled. The intervention proceeded as planned.

**Conclusions:**

For the first time, this pilot trial tests the feasibility and adherence to a multimodal lifestyle intervention, alone or combined with medical food, among individuals with prodromal AD. It can serve as a model for combination therapy trials (non-pharma, nutrition-based and/or pharmacological interventions).

## Introduction

**C**urative or disease modifying treatments for dementia and Alzheimer’s disease (AD) are currently not widely available, and prevention has been identified as a global priority. Estimates have shown that at least 40% of dementia cases are attributable to modifiable vascular and lifestyle-related risk factors (e.g. hypertension, diabetes mellitus, smoking, obesity, physical inactivity, depression, low education), creating a clear window of opportunity for prevention (1). These are consistent with the recent World Health Organization (WHO) Guidelines for risk reduction of cognitive decline and dementia (2). Several randomized controlled trials (RCTs) have tested unimodal interventions for AD/ dementia prevention (e.g. a single drug or single lifestyle intervention, such as cognitive training or physical activity), with negative or very modest results (3).

Recent clinic-neuropathological studies have demonstrated that only 10–30% of clinical AD cases have pure AD pathology, and vascular pathology is the most common comorbidity (4). Thus, unimodal approaches targeting a single pathology are probably insufficient to prevent dementia. Given the multifactorial etiology of late-onset AD, multimodal interventions simultaneously targeting multiple risk factors and disease mechanisms are more likely to be effective. Some recent large-scale European RCTs have tested multimodal preventive interventions in community-dwelling older adults (5). The 2-year Finnish Geriatric Intervention Study to Prevent Cognitive Impairment and Disability (FINGER) (6) (including nutritional guidance, exercise, cognitive training, vascular/metabolic risk management and social stimulation) showed significant benefits on cognition, physical function and health-related quality of life among older adults from the general population, who had an increased risk for dementia (6). In the FINGER trial, at-risk participants were selected using a validated dementia risk score (Cardiovascular Risk Factors, Aging and Dementia - CAIDE Dementia Risk Score) (7). Results from two other multimodal lifestyle trials have also been published; the 6-year Dutch Prevention of Dementia by Intensive Vascular Care (PreDIVA) trial (8) and the 3-year French Multidomain Alzheimer Preventive Trial (MAPT) (9). While these interventions did not have significant effects on the primary outcome, post-hoc analyses suggested potential benefit among individuals with higher dementia risk (in MAPT individuals with a CAIDE score of at least 6 and those with amyloid pathology; in PreDIVA individuals with untreated hypertension). Taken together, these findings further emphasize the importance of targeting individuals with elevated dementia risk (10), and the need for better-defined at-risk groups to achieve an optimal intervention response.

Although patients with prodromal AD (11, 12) represent a group with high risk of progressing to dementia, no effective treatment options are widely available for these individuals. Multimodal lifestyle-based interventions have not been tested in this population. However, the European LipiDiDiet trial (13) investigated the effects of a medical food containing Fortasyn Connect, a combination of nutrients developed to ameliorate synaptic dysfunction. LipiDiDiet was the first multinational RCT to target individuals with prodromal AD identified with the International Working Group 1 (IWG-1) criteria (12). The 2-year intervention did not show significant effects on the primary outcome, i.e. change in cognitive performance measured with a Neuropsychological Test Battery (NTB) composite score, but there were significant beneficial effects on cognitive-functional performance and brain structure (i.e., less cognitive-functional decline and reduction in hippocampal volume (key secondary outcome)) in the intervention group. Longer-term (3-year) intervention benefits were reported on the primary cognitive outcome, and most secondary outcomes, including cognitive-functional and brain atrophy measures (14), which supported beneficial effects of this nutrient combination.

The rationale for combining a lifestyle multimodal intervention with medical food is based on evidence indicating synergistic effects between different intervention components (e.g. omega-3 fatty acids and physical activity) (15–18). This concept has been recently tested in the MAPT trial, where the combined effects of the multidomain lifestyle intervention with omega-3 fatty acid supplementation were assessed among individuals at higher risk for dementia, and led to improved memory score and cognitive status, compared to the placebo (19). Further, nutrient deficiencies in prodromal AD are common (20), and medical food may complement dietary guidance for optimal effects. Building on these results, the Multimodal Preventive Trial for Alzheimer’s Disease (MIND-ADmini) pilot trial was designed as a proof-of-concept trial to assess the feasibility of a multimodal lifestyle intervention, with and without medical food, among individuals with prodromal AD (12).

## Materials and methods

### Study design

**Figure 1:**
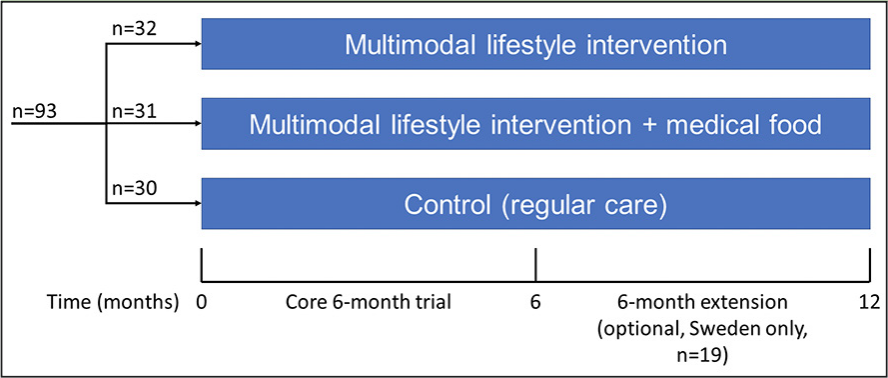
MIND-ADMINI design

The MIND-ADmini (ClinicalTrials.gov identifier NCT03249688) is a 6-month multinational (Sweden, Finland, Germany, and France) proof-of-concept RCT with three parallel groups: 1) multimodal lifestyle intervention; 2) multimodal lifestyle intervention + medical food; and 3) regular health advice/care (control). The trial does not include an arm with medical food alone, as this was investigated in the LipiDiDiet trial (13). Participants were randomized in 1:1:1 ratio in blocks of six (computer generated allocation, two individuals randomly allocated to each group) at each of the four sites after screening by the study nurse. Outcome evaluators were blinded to the randomization group and were not involved in intervention activities. Similar to the FINGER trial, group allocation was not actively disclosed to the participants, and participants were instructed not to discuss the intervention with the outcome evaluators. The study design is summarized in Figure 1. Intervention duration for the core trial was 6 months, and a 6-month optional extension was conducted at the Swedish site.

### Participants

#### Inclusion and exclusion criteria

Screening began on 28 September 2017 and was completed on 21 May 2019. Participants were recruited from memory clinics in Stockholm, Sweden and Toulouse, France; via local media advertisement in Frankfurt Germany; and via the university hospital neurology clinic and previous research cohorts in Kuopio, Finland (i.e., participants who had previously participated in studies and given their consent to be re-contacted for other studies). Inclusion criteria are listed in Table 1.

**Table 1:** MIND-AD trial inclusion criteria

• Age 60–85 years. • Mini-Mental State Examination (MMSE) (21) score of ≥24 • Availability of a study partner	
Criteria for episodic memory disorder defined as −1 SD on 2 out of 8 tests, at least 1 memory	
**Memory**	**Non-memory**
FCSRT - delayed free recall ≤ 8	TMT A ≥ 60
FCSRT free recall - learning ≤ 22	TMT B ≥ 150
WMS-R story delayed recall (%) ≤75%	Symbol Digit Substitution Test ≤ 35 (120 sec.)
WMS-R delayed recall of figures (%) ≤ 75%	Category Fluency ≤ 16 (60 sec.)
Criteria for underlying AD pathology within 1 year prior to screening by either	
CSF β-amyloid (1–42/1–40) *10 ratio <1 and/or total-tau and/or phospho-tau and/or β-amyloid 42 based on local cut-offs OR	
MRI evidence for medial temporal lobe atrophy (MTA score 1 or higher) OR	
Abnormal FDG PET and/or PiB PET compatible with AD type changes	
Lifestyle index: Score of 2 or above required for inclusion. (Each ‘yes’ answer is given one point)	
- Physical activity less than 2.5 hours a week* (defined as physical activity intensive enough to lead to sweating and some breathlessness) - Diet - less than 5 portions* of fruits and vegetables per day - Diet - less than 2 portions* of fish per week - Hypertension (diagnosed by physician or current antihypertensive treatment or SBP>140mmHg or DBP>90 mmHg) - Diabetes (type 1 or 2 diagnosed by physician; or current diabetes medication; or recorded elevated fasting blood glucose or HbA1C as per local guidelines within the past 6 months) - Sleep disturbances, depressive symptoms or psychological stress symptoms, for at least one month, judged by the clinician as having some impact on everyday life.	

*Based on WHO and national guidelines; AD: Alzheimer’s disease; CSF: cerebrospinal fluid; DSB: diastolic blood pressure; FCSRT: Free and Cued Selective Reminding Test; FDG PET: fluorodeoxyglucose (FDG)-positron emission tomography (PET); MTA: medial temporal lobe atrophy; PiB PET: Pittsburgh compound B positron emission tomography; SBP: Systolic blood pressure; TMT: Trail making test; WMS-R: Wechsler Memory Scale-revised.

The IWG-1 criteria were used for prodromal AD, defined as having episodic memory impairment and evidence for underlying AD pathology (12). Episodic memory disorder was defined as -1 SD on at least 2 out of 8 tests, of which at least 1 is a memory test (Table 1). AD pathology was defined as having at least one abnormal cerebrospinal fluid (CSF) or neuroimaging biomarker, both similar to the LipiDiDiet trial (13) (Table 1).

A lifestyle index was used for screening to identify individuals with multiple modifiable vascular or lifestyle-related risk factors and thus potential for improvement. The score was calculated by adding one point for each risk factor (see Table 1). For the last item ‘sleep disturbances, depressive symptoms, or psychological stress symptoms’, having one of these three items was sufficient to receive a score of one point. A total of two points or more were required for inclusion.

Exclusion criteria were: conditions affecting safe engagement in the intervention (i.e., the exercise training program), Dementia according to Diagnostic and Statistical Manual of Mental Disorders, Fourth Edition (DSM-IV), use of omega-3 preparations (> 500mg EPA+DHA per day), alcohol or drug abuse, concomitant severe disease (e.g., recent history of myocardial infarction or cancer), major depressive disorders (DSM-IV), intake of supplements for vitamin B6, B12, folic acid, vitamin C and/or E > 200% of the recommended daily intake unless prescribed by physician, subjects with MRI (or CT) scan consistent with a diagnosis of stroke, intracranial bleeding, mass lesion or normal pressure hydrocephalus. Individuals with an MRI scan demonstrating minimal white matter changes (Fazekas scale for white matter lesions <=3) and up to 1–2 lacunar infarcts that were judged to be clinically insignificant were allowed for inclusion. Severe loss of vision or communicative ability, conditions preventing cooperation as judged by the study physician, concomitant participation in any intervention trial (in the last 30 days) were also exclusion criteria.

#### Intervention program

The multimodal intervention program in MIND-ADmini is based on the FINGER protocol (6), and was adapted for participants with prodromal AD. The FINGER protocol adaptations (Table 2) were made in order to better tailor the intervention to this target population, e.g. adapting the lifestyle intervention schedule to reduce the burden of frequent travel to the trial sites.

**Table 2:** FINGER protocol adaptations made in the delivery, content and evaluation of the intervention program in MIND-ADMINI pilot trial

• Physical exercise sessions were performed in small groups of 4–6 participants.
• Physical exercise training program was conducted when the gym facilities were not occupied by other customers.
• The components of the physical exercise program was performed in 3-week bouts (i.e., 3-w aerobic and 3-w anaerobic) to facilitate adherence/ motivation.
• Physical activity was evaluated with actigraphy for objective measures and to minimize demands of handling exercise diaries.
• The content of the cognitive training program was adapted to the cognitive functioning level of participants with prodromal AD.
• Cognitive training sessions were carried out on site when possible to facilitate adherence.
• Delivery of diet counseling was made in cooperation with study partners to increase adherence when feasible.
• Support for transport to study center was provided if the participant had orientation problems in new areas.

At baseline, the study nurse gave all participants oral and written information and advice on healthy diet and physical, cognitive, and social activities that are beneficial for the management of vascular risk factors and disability prevention. Blood samples were collected from all participants twice during the study, at baseline and 6 months (and at the 12 months visit in the extension in Sweden). In case of clinically relevant abnormal findings from blood results, participants were provided with information and advice to contact primary health care or a referral to primary health care. All participants had a chance to contact the study nurse by telephone or e-mail when needed.

#### The multimodal lifestyle intervention

In addition to general health advice, participants in the two intervention arms (lifestyle and lifestyle+medical food) received the multimodal lifestyle program. The multimodal lifestyle program consisted of: (1) Nutritional guidance; (2) Physical exercise; (3) Cognitive training; (4) Intensive monitoring and management of vascular and metabolic risk factors; (5) Social stimulation. The different intervention components were introduced gradually, and then run in parallel during the whole trial period. The stepwise introduction of intervention components was designed to promote adherence. All intervention components were standardized, ensuring similar intervention content and intensity for all participants at all study centers, while enabling national adaptations and tailoring to individual needs (for examples see Table 2). Social stimulation was integrated into the different intervention domains through group activities that include high levels of social interactions.

#### The nutritional intervention

The nutritional intervention was delivered through 3–4 group sessions (about 60–75 min each) and 3 individual face-to-face counselling sessions (about 30 min each) with the study dietitian. The individual counselling sessions included careful assessment of the participant’s current dietary habits and provided tailored, concrete advice on how to improve diet and implement the changes in their daily routines. The group sessions provided more information, motivation and resources to facilitate lifestyle changes, practical educational activities, while additional support was offered through written information, e.g. tips on food choices, recipes, FAQs. Food frequency questionnaires and 3-day food diaries were administered to monitor diet and dietary changes. Study partners were invited and encouraged to participate in both the individual and group sessions. Participants were advised to consume a diet with the following dietary goals in nutrient intake level that are consistent with New Nordic Recommendations (NNR) 2012 (22) or adapted to the country’s nutritional recommendations (i.e., France and Germany). Participants were advised to consume protein 15–20 E%; total fat 25–40 E%, of which saturated fatty acids <10 E% and trans-fatty acids as low as possible; monounsaturated fatty acids 10–20 E%; Polyunsaturated fatty acids 5–10 E% of which n-3 >1 E%; carbohydrates 45–60 E%, of which refined sugar < 10 E%; dietary fibre 25–35 g/day; salt (NaCl) ≤ 6 g/day; alcohol < 5 E%. These dietary goals are achieved by advising participants to: change from butter and other saturated fats to vegetable fats; consume rapeseed oil, olive oil and vegetable margarines (≥ 60% fat) ≥ 20 g/day; consume fruit and vegetables according to the recommendation (≥ 500 g/day), choose whole grain in all cereal products; limit sucrose intake to ≤ 50 g/day; consume alcoholic beverages at a maximum of 2 units/day for men and 1 unit/day for women. Recommendations were adjusted according to individual needs related to comorbid conditions, medications and body weight. Vitamin D supplementation (10–20 µg/day) (23) was recommended according to each country’s recommendations.

#### The physical exercise training program

The physical exercise program was based on international guidelines (24, 25) and represented a modified version of the FINGER program (6) and the ADEX trial (26). Training was organized in small group sessions which were supervised by a physiotherapist or a personal trainer, preferably 4–5 participants/trainer (26). The exercise program was tailored to participants’ individual physical fitness level and included cardiovascular endurance training and progressive strength training twice a week (about 60 min per session). To facilitate adapting to the exercise program it followed a 3-week bouts design (see Table 3), alternating with resistance exercise and aerobic exercise, both with increasing intensity during the first 12 weeks. During the remaining weeks of the trial, exercise sessions followed a circuit training program alternating strength and aerobic exercises. The strength training program was based on 1 repetition maximum (1RM) and was assessed at baseline and 3 months (additionally at 6 months in the extension study in Sweden), for four main muscle groups (legs, abdomen, back and arms), with a focus on legs and core muscles. Aerobic exercise at the gym was adjusted with the help of the cycle ergometer test results (27) and the Borg scale (28). This was used to ensure that the perceived intensity remains within the right range (not too intense nor too light). Individual aerobic training, such as Nordic walking, cycling and swimming, was encouraged, and all participants were offered advice on how to increase physical activity levels in their daily lives. Additional exercise support was offered through oral and/or written information, e.g. tips on overcoming motivational factors. Participants were monitored for physical activity levels by an accelerometer for one week at baseline, 3 months and 6 months (12 months in the extension study in Sweden) to monitor participants’ progress.

**Table 3:** Description of the MIND-ADMINI exercise program

Average	0–3 mo. (Design: 4 x three weeks bouts to accustom to exercising)	4–6 mo.
Resistance exercise	Group training 4–5 participants/ PT or individual exercise	Group training 4–5 participants/ PT or individual exercise
Exercise frequency/w	2	2
Duration of exercise/minutes	60	60
Number of muscle groups	4–8 (primarily legs/ core muscles)	8–10
Repetition/ set	8–15	10–20
Load %1 RM *	Bout 1: .40–50 Bout 3: 60–80	60–80
Number of sets	2	2–3
Aerobic exercise Group training 4–5 participants/PT	Bout 2: 50–60% max HR (15–17 RPE) Bout 4: 60–70% max HR (16–18 RPE)	Moderate to high intensity 70–80% max HR (17–19 RPE)

* RM = Repetition Maximum. 1 RM corresponds to the highest load that can be lifted through the entire range of motion once

#### Cognitive training

Cognitive training included group and individual sessions. The 2–3 group sessions (duration approximately 60–75 min per session) were led by psychologists or occupational therapists and involved: general information about neurocognitive disorders, coping and reasoning strategies, introducing the cognitive training program and instructing its use. An additional group session was added in the 6-months extension (in Sweden). Individual training sessions consisted of computer-based training at home or together with other participants, each with their own computer, at the study site (twice a week, approximately 15–30 min per session). The cognitive training program is a web-based, in-house developed computer program including several tasks adapted from protocols previously used in the FINGER trial (6). The program consisted of eight tasks and targeted four cognitive functions: executive processes (updating spatial locations, updating numbers, and a mental set-shifting task), working memory (a spatial maintenance task), episodic memory (relational and spatial memory tasks), and mental speed (odd-even and high-low number tasks). As in the original FINGER trial, the program automatically adjusted the level of task difficulty for all tasks (except the shifting task) to the individual performance, by a requirement of 80% success rate for the next difficult level. The tasks were arranged in two blocks (A and B) and were practiced every other session. This program was adapted in MIND-ADmini by the addition of a less demanding entry level in the updating tasks. The participants were encouraged to perform the cognitive training program at home, and they were provided with individual log-in accounts and instructions for using the web-based program. They were also encouraged to contact the psychologist to receive any support they needed to manage their training from home.

#### Social stimulation

Social activities were stimulated through the numerous group meetings within the intervention domains (physical exercise, diet, and cognitive training). The group sessions were designed to facilitate open discussions and interactions with other participants. One example is the physical exercise sessions which were performed twice a week in the same small groups throughout the intervention period. After each training exercise session, participants were offered a healthy snack and had the opportunity to freely discuss any aspects related to physical activity with each other and the trainer. Furthermore, study partners were encouraged to participate in all sessions.

#### Management of metabolic and vascular risk factors

Management of metabolic and vascular risk factors was based on national evidence-based guidelines (29). It included one additional meeting with the study nurse (at 3 months), for measurements of blood pressure, weight and BMI, hip and waist circumference, and further recommendations for lifestyle management and to enhance motivation. If medication initiation or adjustments were needed, the study physician either wrote a prescription or referred the participant to regular healthcare, as appropriate according to local procedures. Smokers were also provided with support to quit smoking.

## The medical food intervention

The lifestyle + medical food intervention group received all the above-mentioned lifestyle intervention components and in addition the study product Fortasyn Connect, a 125ml once-a-day milk-based drink. The specific multi-nutrient combination Fortasyn Connect contains long-chain omega-3 fatty acids docosahexaenoic acid (DHA) and eicosapentaenoic acid (EPA), uridine monophosphate, choline, vitamins B12, B6, C, E, and folic acid, phospholipids, and selenium. This combination of nutrients was designed to enhance efficacy over what can be achieved by individual nutrients. For example, it synergistically enhances the formation of neuronal membranes by providing rate limiting compounds for the synthesis of brain phospholipids, and targets AD-related pathological processes. In Sweden, Finland, and Germany Fortasyn Connect (Souvenaid™) is available on the market in 125ml bottles. Danone Nutricia Research provided this product for the MIND-ADmini trial.

## Follow-up and outcome measurements

All participants (control and intervention groups) met the study nurse at screening, baseline, and at 6 months (additionally at 12 months in the extension study in Sweden) for measurements of blood pressure, weight and BMI, and hip and waist circumference. All participants (control and intervention groups) met the study physician at screening and at 6 months for a detailed medical history and physical examination. At baseline and at 6 months, the cognitive status of the participants was assessed by a psychologist, and information on health status, lifestyle, demographic and socioeconomic factors was collected.

The primary outcome of the MIND-ADmini pilot trial is feasibility of the multimodal intervention measured by:

1. *Recruitment rate* defined as proportion of participants who are randomized of those who fulfilled the criteria and were invited to participate during a 6-month recruitment phase. We consider a recruitment rate of 50% or more as successful.
2. *Overall adherence to the intervention* measured in each of the two intervention arms (composite measure of participation in different intervention components). Several measures of adherence will be used: for cognitive training, attendance at the cognitive training sessions including automatic recordings of computer program use, and group sessions attendance; for physical exercise, participation in exercise sessions; for nutrition, attendance at the group and individual nutrition sessions; for vascular care, attendance at the 3-month follow-up visit with the study nurse, where measurements are taken for blood pressure, weight, BMI, hip and waist circumference; for medical food, consumption of medical food product measured through a study product diary, similar to LipiDiDiet trial. If a participant attends a minimum of 40% of sessions per domain, in at least 2/4 domains (exercise, nutrition, cognitive training and monitoring of vascular/metabolic risk factors), the participant is considered to successfully adhere to the lifestyle intervention. In the lifestyle + medical food intervention arm, the participant is considered to successfully adhere to the intervention if they in addition consume at least 60% of the medical food study product.
3. *Retention rate* defined as proportion of participants who complete the 6-month trial period. We consider a successful rate if less than 35% of participants drop out. The reason for drop-out will be recorded as provided by the participant.

Secondary outcomes are:

1. *Adherence to intervention components* in each of the two intervention arms i.e. adherence to exercise (attendance at the exercise sessions), nutritional guidance (attendance at the nutrition sessions), cognitive training (including attendance at the group sessions and cognitive training sessions), intensive monitoring of vascular/ metabolic risk factors (session attendance), and medical food adherence (study product diary similar to LipiDiDiet trial).
2. *Adherence to healthy lifestyle changes*, monitored with the composite healthy lifestyle score, which will be based on several components and calculated for all trial participants. The healthy lifestyle score will be based on several components. For the exercise component, short physical activity questionnaire (frequency and duration of activity, Swedish National Board of Health and Welfare (30)) will be used, and objective measurements (e.g. actigraph) will also be considered. For nutritional guidance, the HATICE food questionnaire (measure from the European diabetes projects ePredice trial) is used. Cognitive activities are measured through questions regarding participation/engagement in cognitively stimulating activities. For vascular/metabolic risk factors, adherence to national guidelines is assessed. Measures used in the original FINGER trial will also be considered.

Exploratory outcomes are: changes in vascular and metabolic risk factors; depressive symptoms (Geriatric Depression Scale) (31); stress-related symptoms (Perceived Stress Scale) (32); physical performance; timed 10-meter walking test; timed 10-meter dual-task test (33); 30 second chair stand test and Short Physical Performance Battery (SPPB) (34); health-related quality of life (RAND-36) (35); Insomnia Severity Index (36); blood biomarkers (e.g. lipids, markers for inflammation, vitamins D & B); experiences of the participants (qualitative data based on interviews).

Cognition was measured through a detailed cognitive assessment (Neuropsychological Test Battery, NTB) and functional assessments (Clinical Dementia Rating, CDR (37) and CDR sum of boxes; and Alzheimer’s Disease Cooperative Study-Activities of Daily Living, ADCS-ADL (38)) at baseline and at the 6-month visit to obtain reliable estimates of change over time for power calculations for a future larger multimodal intervention trial based on the current pilot trial.

### Statistical considerations

The MIND-ADmini trial was expected to include up to 150 participants. The recent CONSORT extension guidelines for developing and reporting pilot RCTs state that “no hypothesis testing is recommended” for pilot trials (39). No formal sample size calculations were needed because the primary outcome measures are feasibility (recruitment and intervention acceptability - i.e., adherence and retention), safety and adherence to the multimodal intervention in participants with prodromal AD. All analyses will be conducted at the group level. The three trial arms will be compared to assess the differences in the primary, secondary and exploratory outcomes.

### Ethics and safety aspects

MIND-ADmini was approved by local ethical committees in all four countries. Participants and study partners gave their written informed consent prior to enrollment in the trial. Additional informed consent was obtained before participation in the optional 6-month extension study. The trial adhered to the Declaration of Helsinki and is conducted according to the International Conference on Harmonisation (ICH) guidelines for Good Clinical Practice (GCP). Study participants were insured according to local legislation. Participants had the possibility to contact the study staff throughout the study and they were referred to appropriate medical care when indicated. The lifestyle intervention protocol was adjusted for the prodromal AD target group. The safety of the intervention was assessed, and data were collected on adverse events (AEs) and serious adverse events (SAEs).

Data management process: A logistic system to schedule appointments was created and designed by the MIND-AD Consortium. Form templates and data collection files were electronically provided by the trial coordinators, and study forms were printed at each center. For validated scales, already available translated versions were used. Other documents were translated at the local trial centers. Source data and other data collection forms were entered in an electronic case report form (eCRF) using the SMART-TRIAL platform (40). Monitors verified the completed CRFs against the source data and trial database. The Investigator Site File was regularly updated according to ICH-GCP guidelines.

### Study progress

The study started in September 2017 in Sweden, November 2017 in Finland, April 2018 in Germany and October 2018 in France. In total, 93 participants were randomized, and the 6-month intervention was completed in December 2019. Final data checks and database lock were delayed due to Covid-19 pandemic restrictions. Data analysis is ongoing.

Baseline characteristics of the participants are summarized in Table 4. The mean (SD) age of the entire sample was 72.9 years, level of education was 12.8 years, and MMSE score 27.7 points. Vascular and lifestyle-related risk factors were common as expected, based on the inclusion criteria: Impaired fasting glucose (≥ 6.1 mmol/L) was seen in 21%, and 57% were overweight, with BMI ≥25 and, 13% had BMI ≥30. The baseline characteristics of participants are presented in Table 4.

**Table 4:** Preliminary baseline characteristics of the trial participant

**N=93**	**Baseline**
Age (years)	72.9 (6.2)
Women, N (%)	48 (51.6%)
Education (years)	12.8 (3.6)
MMSE	27.7 (1.6)
SPB (mmHg)*	142.1 (17.2)
DBP (mmHg)*	81.0 (9.7)
LDL-C (mmol/L)	3.1 (1.3)
HDL-C (mmol/L)	1.6 (0.5)
BMI (kg/m2)	25.9 (3.7)
Fasting glucose (mmol/L)	5.8 (0.6)
Physical activity less than 2.5 hours a week, N (%)	41 (44%)
Diet - less than 5 portions of fruits and vegetables per day, N (%)	64 (69%)
Diet - less than 2 portions of fish per week, N (%)	58 (62%)
Depressive symptoms	33 (35%)
High blood pressure	49 (53%)
Diabetes	8 (9%)
Sleep disturbance	38 (41%)
Feeling stressed	33 (35%)

* Data not available for 26 participants. Characteristics are calculated based on number of participants with available data. Numbers are means (SD) unless otherwise specified.

## Discussion

The MIND-ADmini trial investigates whether a multimodal lifestyle-based intervention is feasible among individuals with prodromal AD in four European countries (12). Finding effective and feasible prevention strategies is particularly important for individuals with prodromal AD who currently lack effective treatments. While multimodal lifestyle interventions have been indicated to be efficient and feasible among individuals at risk for dementia, their feasibility and effects have not been studied in RCT settings among individuals who already have cognitive impairment. Knowledge is needed about the required dosage and frequency of the multimodal intervention and whether adaptations of the intervention components (e.g. nutrition, exercise, cognitive training) can ensure adherence and optimize the benefits.

Multidomain RCTs for dementia prevention and risk reduction have been adopting novel designs and implementation strategies. The MAPT trial for example combined omega 3 polyunsaturated fatty acid supplementation with a multimodal lifestyle intervention (nutritional advice, physical activity and cognitive training), and tested their effects alone or in combination among older adults with memory complaints (9). Similarly, the MIND-ADmini tested a multimodal lifestyle intervention alone or combined with medical food (Fortasyn Connect). This is an important step towards combining pharmacological and non-pharmacological interventions. The MIND-ADmini trial builds on previous experiences from multimodal prevention trials, and incorporates recent suggestions for prevention interventions to target at-risk individuals, as opposed to non-selected groups (41). Further, MIND-ADmini will contribute to important methodological advancements in prevention trials, as it will collect feedback on participants’ experiences through qualitative interviews. Experiences from MIND-ADmini will aid in designing larger efficacy trials, as well as future large international multimodal trials among at-risk populations.

In current AD research, there is an increasing emphasis on early interventions, e.g. for individuals in an early-symptomatic disease stage such as prodromal AD. In MIND-ADmini the IWG-1 criteria for prodromal AD (12) were used, even though prodromal AD can now be defined using newer research diagnostic criteria (11, 42). One reason for using the IWG-1 criteria is that they are more pragmatic and easier to apply in multinational settings. This is mainly because there is more flexibility in terms of which biomarkers can be tested, and CSF is not required. Also, these criteria were used in the LipiDiDiet trial (13, 14). Using these IWG-1 criteria in LipiDiDiet, 88% of participants (with centrally analyzed CSF available) were confirmed to be amyloid positive, meaning that these criteria performed well at identifying a prodromal AD population with underlying amyloid pathology (43).

To further advance multidomain lifestyle intervention trials for dementia risk reduction, the World-Wide FINGERS (WW-FINGERS) network was launched in 2017 (44). WW-FINGERS is the first global network of multidomain lifestyle intervention trials for dementia risk reduction and prevention, including over 35 countries in different geographical, cultural and economic settings (45). WW-FINGERS aims to harmonize and adapt multidomain interventions across various countries and settings, facilitate data sharing and analyses across studies, and promote international joint initiatives, to identify globally implementable and effective preventive strategies. WW-FINGERS will also create a unique opportunity for rapid knowledge dissemination and implementation, and the MIND-ADmini trial will contribute to this as a valuable resource since it serves as a model for combination therapy trials (non-pharma, nutrition-based and/or pharmacological interventions).
